# Supplementary material for: Performance Assessment of Self-Healing Polymer-Modified Bitumens by Evaluating the Suitability of Current Failure Definition, Failure Criterion, and Fatigue-Restoration Criteria
Source: Materials (Basel). 2023 Mar 21;16(6):2488. doi: 10.3390/ma16062488 (PMC10059980; doi:10.3390/ma16062488)
Supplement: Supplementary file 1 [file materials-16-02488-s001.zip › materials-2259895-supplementary.pdf]

Supplementary files

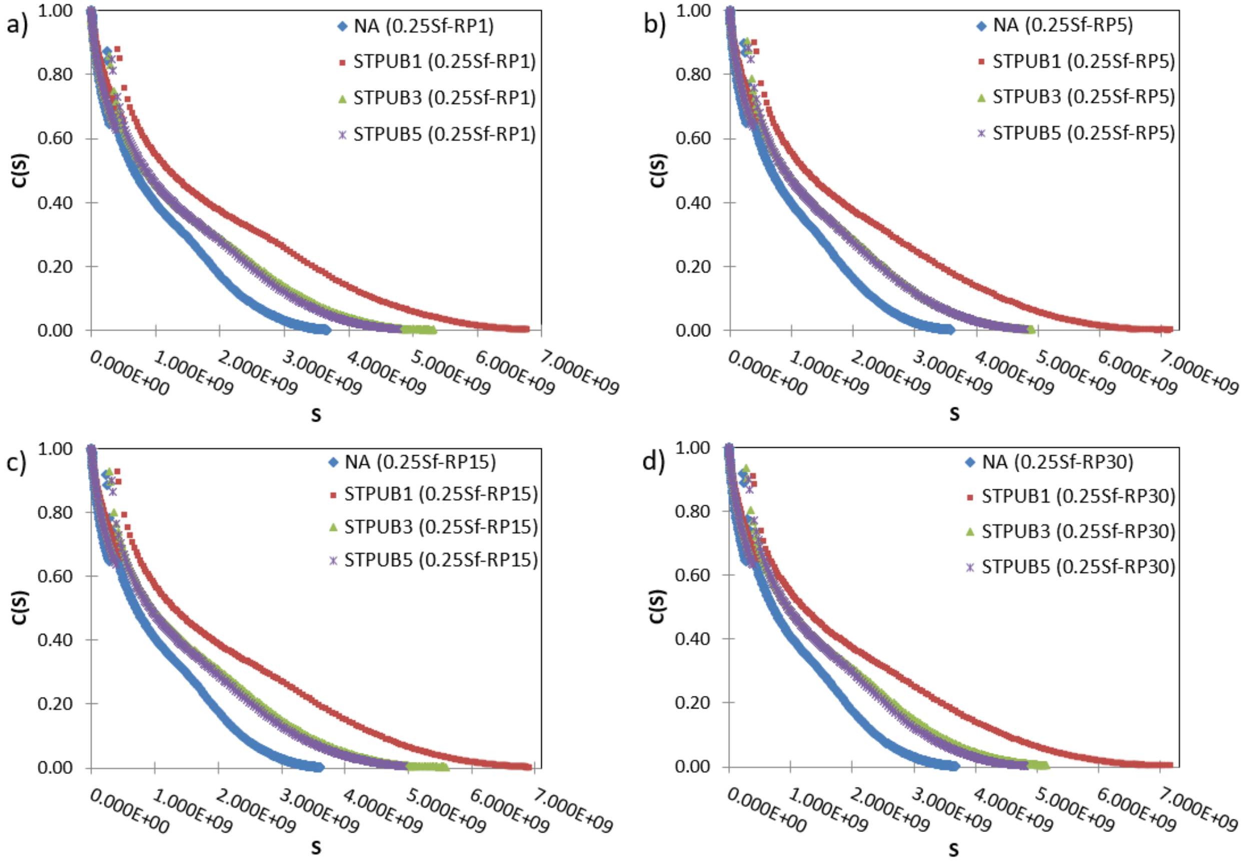

Figure S1. DCCs related to all bitumens at 25% of S_f: (**a**) RP1; (**b**) RP5; (**c**) RP15; (**d**) RP30.

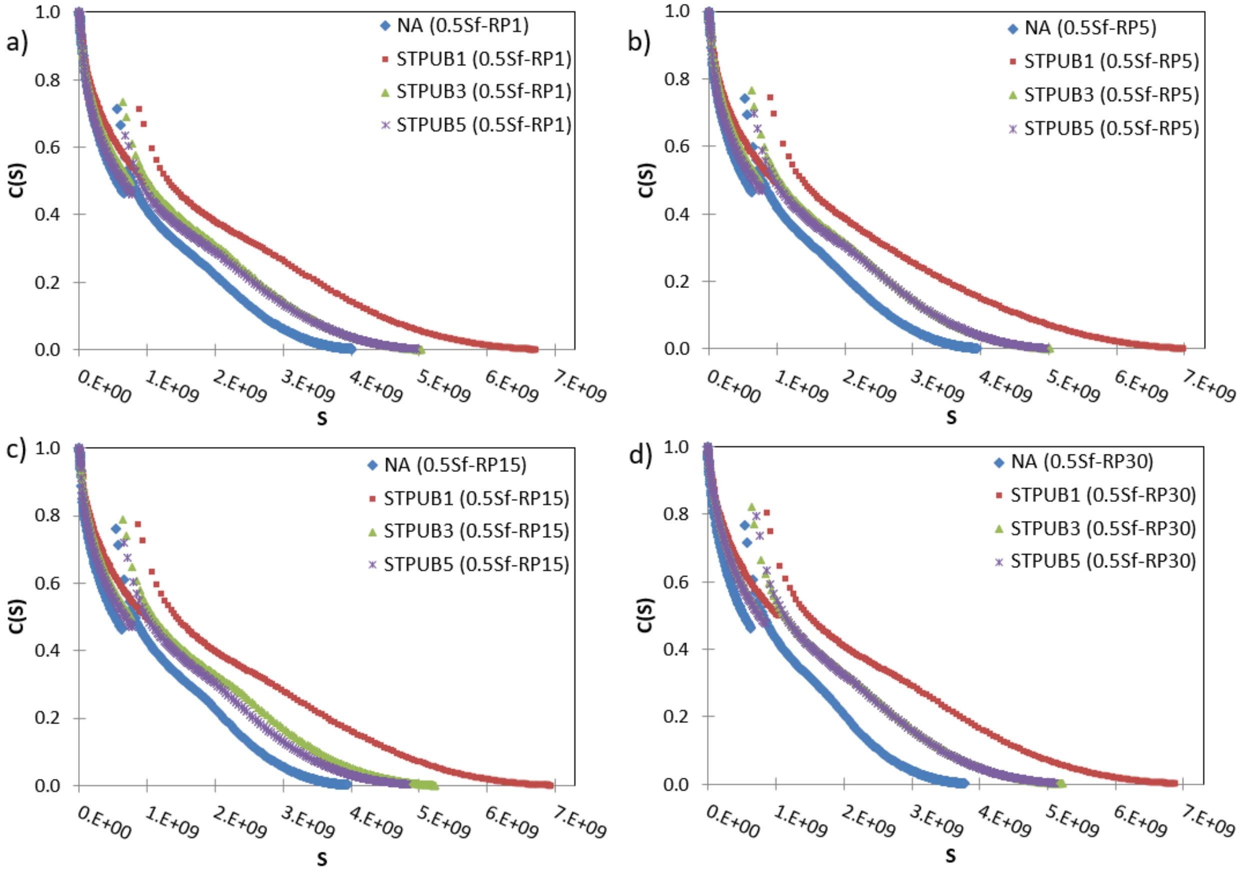

Figure S2. DCCs related to all bitumens at 50% of S_f: (**a**) RP1; (**b**) RP5; (**c**) RP15; (**d**) RP30.

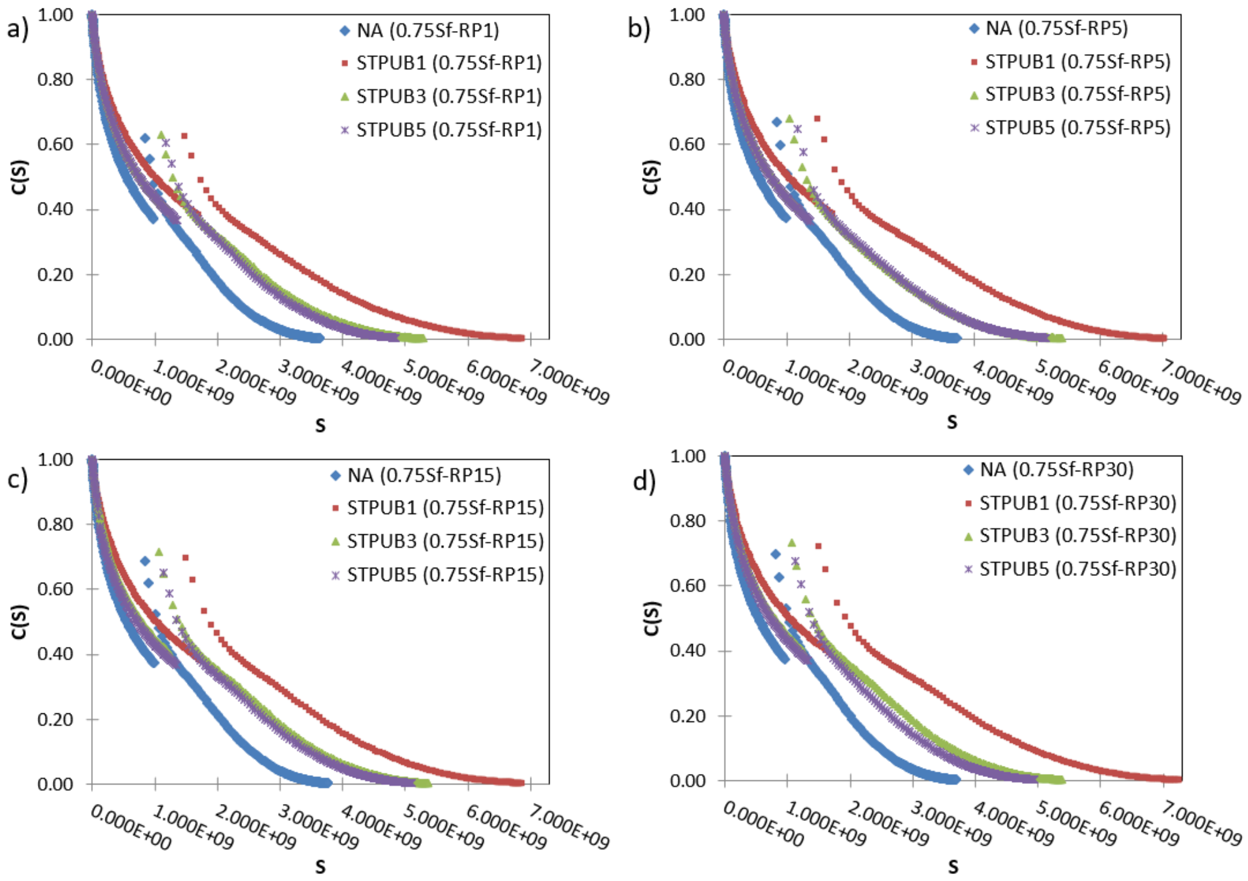

Figure S3. DCCs related to all bitumens at 75% of S_f: (**a**) RP1; (**b**) RP5; (**c**) RP15; (**d**) RP30.

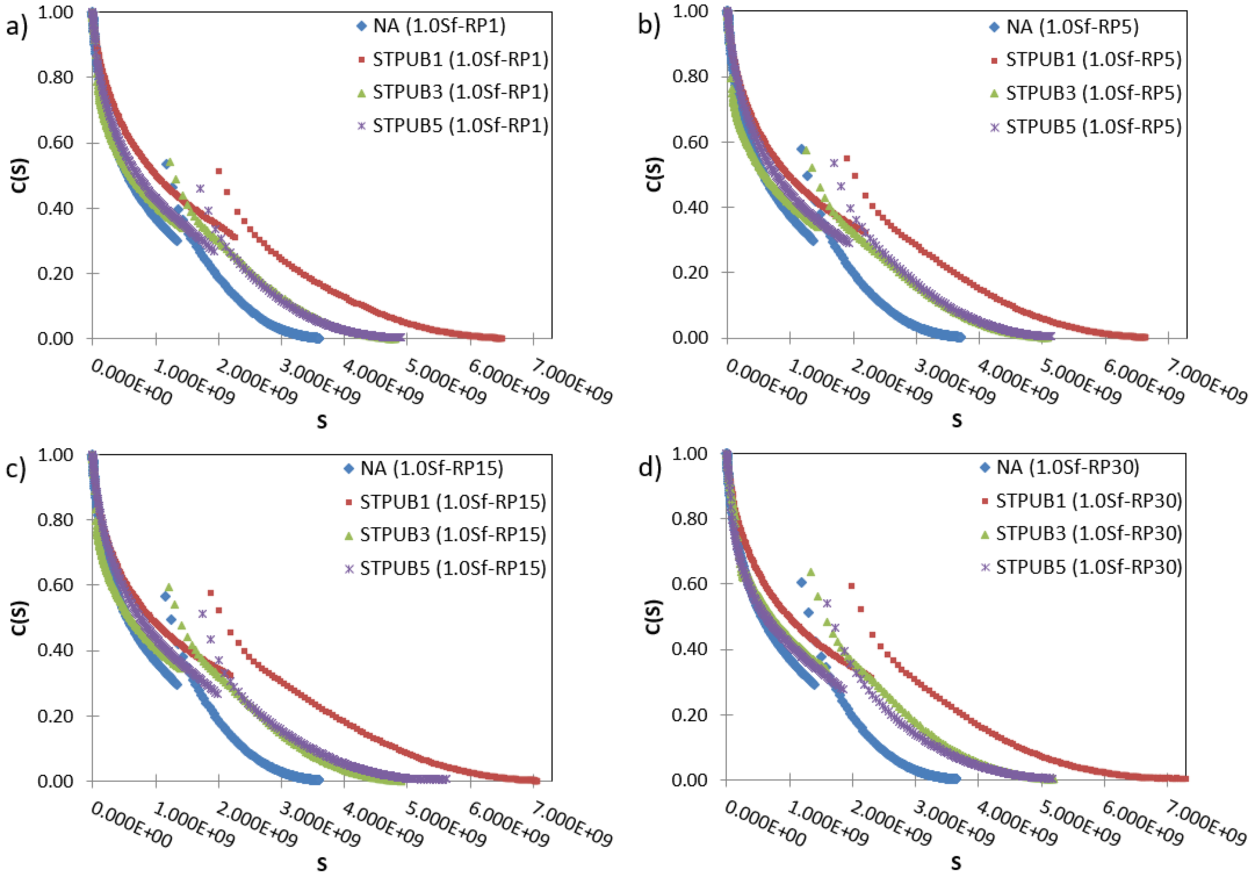

Figure S4. DCCs related to all bitumens at100% of S_f: (**a**) RP1; (**b**) RP5; (**c**) RP15; (**d**) RP30.

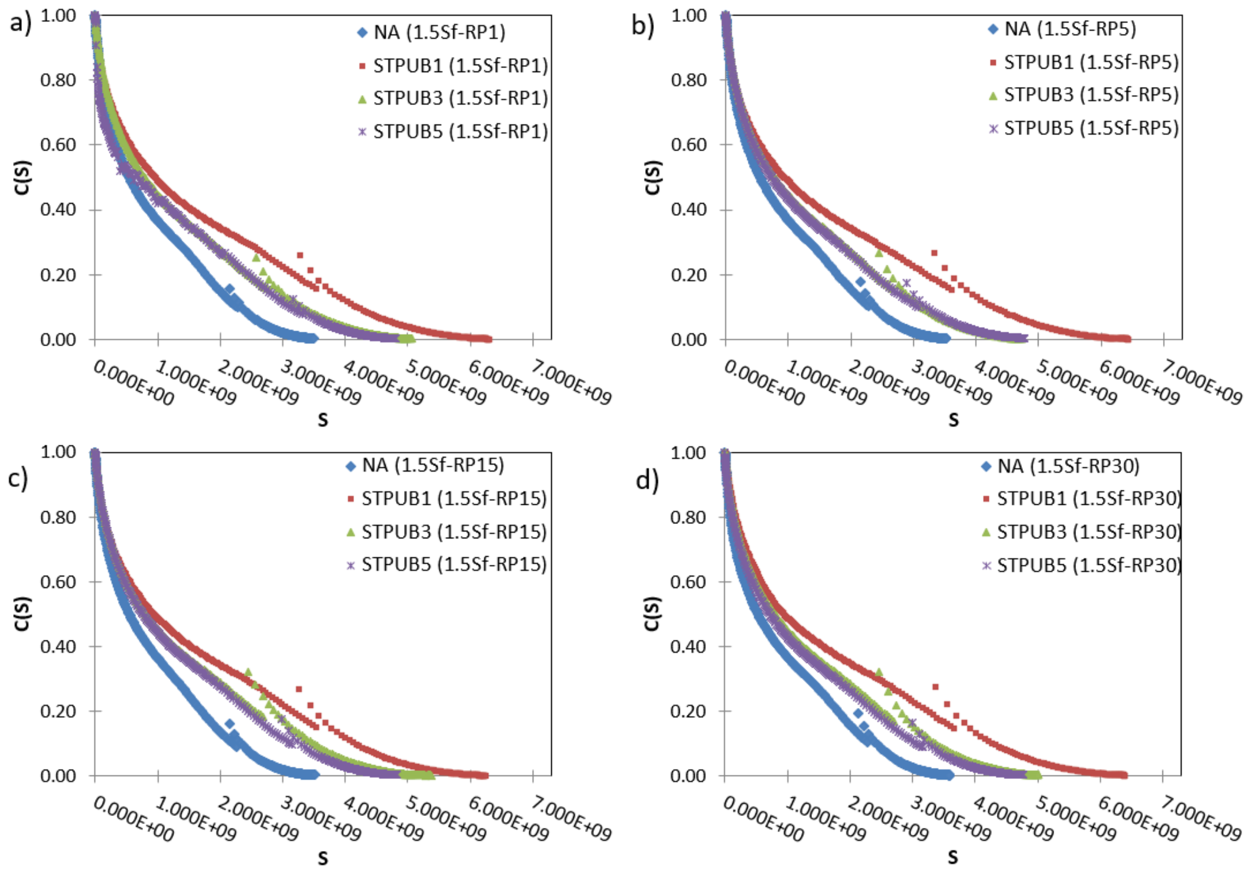

Figure S5. DCCs related to all bitumens at150% of S_f: (**a**) RP1; (**b**) RP5; (**c**) RP15; (**d**) RP30.
